# Celebrating the 130th anniversary of Wuhan University

**DOI:** 10.1039/d3sc90244g

**Published:** 2024-01-17

**Authors:** Lin Zhuang, Qiu Wang, Aiwen Lei, Qianghui Zhou

**Affiliations:** a College of Chemistry and Molecular Sciences, Wuhan University Wuhan China lzhuang@whu.edu.cn aiwenlei@whu.edu.cn qhzhou@whu.edu.cn; b Duke University USA qiu.wang@duke.edu

## Abstract

Lin Zhuang, Qiu Wang, Aiwen Lei and Qianghui Zhou introduce the *Chemical Science* and *Green Chemistry* joint themed collection celebrating the 130th Anniversary of Wuhan University.
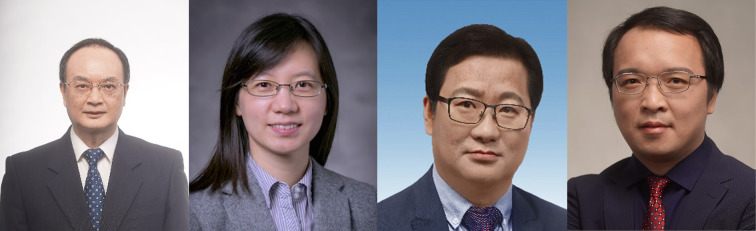

Founded in 1893 as the Ziqiang Institute and renamed the National Wuhan University in 1928, Wuhan University (WHU) is a comprehensive research university which has nurtured notable alumni making key contributions to the development of natural sciences, galvanized by its motto *Self-improvement*, *Perseverance*, *Truth-seeking*, *Innovation*.

Inaugurated as the Department of Chemistry under the Science–Engineering College of the then National Wuhan University in 1928, the current College of Chemistry and Molecular Sciences at WHU was established in January 2001. Recent years have witnessed extensive developments in chemistry at WHU, with the College now consisting of eight departments as well as four key laboratories. In 2017, the subject chemistry was selected by the Ministry of Education of China as one of the key construction disciplines of the national “Plan for the Construction of Globally-leading Universities and First-class Disciplines” as one of the ten disciplines at WHU.

As the university celebrates the 130th Anniversary this year, in observance of this special occasion, the Royal Society of Chemistry is proud to present this joint themed collection collated between *Chemical Science* and *Green Chemistry*, on which over 180 articles have been published by researchers from WHU so far. This themed collection includes contributions from WHU scholars, alumni and collaborators conducting research in energy, green chemistry, chemical biology, *etc.*, encompassing all areas of chemistry. It is with pleasure to feature some of the articles from the collection in this editorial.

## Novel enabling synthetic methodology

Researching *metal*- and photo-catalyzed methods to promote S_N_Ar reactions between thiols and aryl halides, Dawei Ma and coworkers, report an S_N_Ar reaction of thiols with most electron-poor aryl halides taking place at room temperature under the action of K_2_CO_3_ and DMAc (http://doi.org/10.1039/D3GC02066E). Critically, two more powerful oxalic diamide ligands were identified to facilitate the Cu-catalyzed coupling reactions with low catalytic loadings. Jin-Quan Yu, Zhong Jin and coworkers develop an efficient macrocyclization strategy *via* Pd-catalysed intramolecular *meta*-C–H olefination using a practical indolyl template. This approach has been successfully employed to access macrolides and coumarins (https://doi.org/10.1039/D3SC01670F). Xiao-Feng Wu and coworkers, develop a two-step protocol to access 1,2-diarylethanones, involving site-selective C(sp^2^)–H thianthrenation and carbonylation of arenes under mild conditions (https://doi.org/10.1039/D3SC02402D). Jinshuai Song, Hai-Chao Xu and coworkers, develop a photoelectrocatalytic C–H amination approach for synthesizing anilides *via* the C–H/N–H coupling of arenes and carbamates (https://doi.org/10.1039/D3GC02126B). Jing-Hao Qin, Chong-Hui Xu, Yang Li and Jin-Heng Li and coworkers, present an elegant rhodium-catalyzed electrochemical [2 + 2 + 2] cyclotrimerization of 1,3-butadiynes, showcasing a method for the regioselective synthesis of structurally diverse hexasubstituted arenes (https://doi.org/10.1039/D3GC02831C). Dengke Ma and Youai Qiu and coworkers, report an electrochemical nickel-catalysed defluoroalkylation of *gem*-difluoroalkenes with alkyl halides, enabling the synthesis of monofluoroalkene products (https://doi.org/10.1039/D3GC02814C). Yi-Hung Chen, Xinxing Zhang, Aiwen Lei and Hong Yi and coworkers, report the identification and reactivity elucidation of the fragile intermediates in electrochemical oxidative α-C(sp^3^)–H functionalization of tertiary amines (https://doi.org/10.1039/D3SC00527E). Chunlan Song, Jiakun Li and coworkers, report an electrochemical ring-opening 1,3-dihydroxylation of arylcyclopropanes with H_2_O, enabling the direct formation of 1,3-diols through controlled electrochemical C–C bond cleavage of arylcyclopropanes with H_2_O as the ultimately green hydroxyl source (https://doi.org/10.1039/D3GC02283H).

## Sustainable and green chemistry

Qixue Qin and Ning Jiao and coworkers, present a novel and efficient approach for the oxidative rearrangement of cyclobutenones, by utilizing I_2_ as a catalyst and dimethyl sulfoxide (DMSO) as a greener oxidant and source of oxygen (https://doi.org/10.1039/D3GC01756G). Si-Shun Yan, Yong-Yuan Gui and Da-Gang Yu, and coworkers, report a photocatalytic defluorocarboxylation using formate salts as both a reductant and a carbon dioxide source. This transition metal-free strategy provides a mild, efficient, and sustainable approach for accessing a series of valuable aryl acetic acids, such as flurbiprofen (https://doi.org/10.1039/D3GC01299A). Chen-Guo Feng, Yingbin Liu, Guo-Qiang Lin and coworkers, report a convenient method for synthesizing aryl-containing trisubstituted alkenes through direct alkylation of alkenes under solvent-free and catalyst-free conditions (https://doi.org/10.1039/D3GC02685J). Liyan Song, Ran Lin and Rongbiao Tong and coworkers, report the inaugural green oxidation of amides and aldehydes (alongside NaN_3_) using oxone and halide to yield the *N*-halo amides and acyl azides, the key intermediates for Hofmann and Curtius rearrangements respectively (https://doi.org/10.1039/D3GC04355J).

## Electrochemical energy storage and conversion

Li Xiao, Lin Zhuang and Héctor D. Abruña, and co-workers, found that the degradation of the ionomer in an anion exchange membrane fuel cell (AEMFC) is considerably more severe than that of the membrane under realistic operation (https://doi.org/10.1039/D3SC03649A). Wei Luo and co-workers, found that a sulphate functionalized Ru catalyst (Ru–SO_4_) exhibited remarkable electrocatalytic performance and stability toward alkaline hydrogen oxidation reaction (https://doi.org/10.1039/D3SC02144K). Huayi Yin and co-workers, systematically summarize the recycling methods using salts as agents to enable the conversion of cathode materials of spent lithium-ion batteries (LIBs), in a review article (https://doi.org/10.1039/D2GC04620B). Jun Huang and Shengli Chen and co-workers, reveal that electrostatic interactions play a decisive role in coupled charge transfer and Li+ segregation in the LiFePO_4_ cathode material (https://doi.org/10.1039/D3SC04297A). Jiangfeng Qian and co-workers, propose a phenazine-mediated chemical sodiation strategy to precisely synthesize a Na-enriched Na_4_V_2_(PO_4_)_3_ cathode under the guidance of a redox-potential-matching principle, and significantly boost the energy density of sodium-ion batteries (SIBs) (https://doi.org/10.1039/D3SC03498D). Xiangjun Pu, Mingyue Ding and Zhongxue Chen and coworkers, propose delta-oxovanadium phosphate as the cathode material for aqueous zinc-ion batteries (https://doi.org/10.1039/D3SC02382F). Lianhuan Han and co-workers, demonstrate a controllable electrochemical approach to modulate the defect density and tune the electrochemical activity of single-layer graphene (https://doi.org/10.1039/D3SC03920J).

## Synthesis and studies of functional molecules: impacts on biology, drug discovery, and the environment

In molecular imaging, recognition elements for various targets play a crucial role in high-performance imaging. The review by Bingqian Lin and coworkers, provide a comprehensive summary of engineered aptamers for molecular imaging, including various design strategies, a wide range of targets ranging from cell membrane proteins, nucleic acids, organelles, metabolites, to metal ions, as well as different imaging modalities (https://doi.org/10.1039/D3SC03989G). In an effort to develop dehydroalanine (Dha)-modified strategies, Yanmei Li and colleagues, report a photoinitiated 1,3-dipolar cycloaddition reaction between Dha and 2,5-diaryl tetrazoles (https://doi.org/10.1039/D3SC02818F). The reaction they worked on exhibited complete site-specificity in the modification of thiostrepton in high yields, providing a chemoselective approach for precise functionalization of proteins. With fluorogenic properties, the reported photo-controllable methodology could be applied to live cell imaging, thus expanding the practicality of the Dha modification methodology. Yanfeng Dang, Xiu-Qin Dong and Chun-Jiang Wang and coworkers, report a new strategy that enables, for the first time, the atroposelective synthesis of axially chiral *N*,*N*′*-*pyrrolylindoles *via de novo* indole formation catalyzed by chiral phosphoric acid (CPA). This work also provides a preliminary investigation on the biological activity of these *N*,*N*′-pyrrolylindoles and their low IC_50_ value toward different cancer cell lines suggests their encouraging potentials in drug discovery (https://doi.org/10.1039/D3SC03686C). Xiaoguang Lei and coworkers, disclose the first total syntheses of a family of alkaloids, including picrasidines G, S, R, and T and related derivatives, in a concise manner. The success of this work is attributed to an innovative late-stage aza-[4 + 2] cycloaddition, which also provides synthetic evidence for the proposed biosynthetic pathway of ITHQ-type bis-β-carboline alkaloids that have appealing biological activities (https://doi.org/10.1039/D3SC03722C). Twisted polyarenes with persistent chirality are desirable but their synthesis has remained a challenge. Zhe Sun and Jishan Wu and coworkers, report highly twisted 1,2:8,9-dibenzozethrene and its vertically fused dimers and trimers by a “one-pot” nickel-catalyzed cyclo-oligomerization reaction. This study also offers an efficient way to access highly twisted polyarenes with enantiomers that can be separated with either left-handed or right-handed helicity (https://doi.org/10.1039/D3SC02285D). Radiocesium ions are a major species in radioactive wastewater, therefore, efficient treatment and isolation methods for Cs ions are highly sought. Xiaodong Shi and coworkers, report the preparation of a covalently tethered isoguanosine self-assembled pentamer that could selectively extract Cs ions with good reusability. This new extractor served as a new platform for the treatment of radiocesium in an environmentally benign manner (https://doi.org/10.1039/D3GC02932H).

This collection continues to amass new and exciting research as more articles are being accepted from WHU researchers and associates. We wish WHU a happy anniversary and hope our readers have a good time reading the collection.

## Supplementary Material

